# Strategies for conducting health research with Latinos during times of political incivility

**DOI:** 10.1002/nop2.166

**Published:** 2018-06-19

**Authors:** Rayna Sage, Sandra Benavides‐Vaello, Erin Flores, Sydnie LaValley, Patrick Martyak

**Affiliations:** ^1^ University of Montana Rural Institute for Inclusive Communities Missoula MT USA; ^2^ Montana State University College of Nursing Bozeman MT USA

**Keywords:** Latinos, participation, political incivility, recruitment, retention

## Abstract

**Aim:**

The current US political climate towards immigrants introduces new challenges for researchers already struggling to recruit and retain Latino participants in health research. The purpose of this work is to illuminate how current political incivility further deters participation by Latinos in research and present strategies to overcome these challenges.

**Design/Methods:**

In this discursive piece, we describe how political incivility serves as a proxy for discrimination, discusses the impact of political incivility on health and health outcomes and explores existing knowledge of recruitment and retention strategies in the light of a current, adversely impacted health study with Latinos during the 2016 election season.

**Results:**

Future work should consider the critical contextual factors (including political incivility) faced by Latinos in the US, while continuing to engage in established research strategies such as increasing trust, developing strong community presence, reducing risks (real and perceived) and being thoughtful in research design and implementation.

## 1 INTRODUCTION

Social determinants of health are the environmental conditions where people are born, grow, live, work and age (Centers for Disease Control and Prevention, 2014). Conditions, such as discrimination, poverty, unsafe communities and limited resources, have an impact on health, health outcomes, quality of life and often yield health disparities for certain populations, including low‐income US Latinos. Consequently, Latinos are more likely to experience worse health outcomes particularly in relation to chronic conditions such as diabetes and hypertension (Nelson, 2002).

Undoubtedly, research is a key to reducing the health disparities. However, recruiting Latinos and other minority groups, particularly those of low socioeconomic status (SES), remains a challenge for most health researchers (Freedman et al., 1995; Nicholson, Schwirian, & Groner, 2015). These groups are less likely to be recruited into research studies and consequently remain underrepresented in research, including clinical trials, epidemiological inquiries and intervention development (Clark, 2012; Ford et al., 2013; Hébert et al., 2015; Nelson, 2002; Rogers & Lange, 2013; Yancey, Ortega, & Kumanyika, 2006). This discursive piece provides an overview of key reasons for the low participation of Latinos in health research and summarizes existing strategies that are key to the recruitment and retention of this demographic in research inquiries.

There are myriad reasons for why Latinos of low SES are less likely to participate in research studies. These include a lack of understanding of the research process, the importance of participation, distrust of institutions, fear of discrimination or the actual experience of discrimination (Ejiogu et al., 2011; Free, Hoile, Robertson, & Knight, 2010; McDonald et al., 2011; Rosal et al., 2010). The latter, discrimination, is one of the most caustic determinants and has the capacity to have an impact on a broad array of health concerns. These may vary from increased risk for mental health conditions, such as anxiety and depression, to poor physical health outcomes such as hypertension (Pascoe & Smart Richman, 2009).

The current US political climate towards immigrants introduces new challenges for researchers already struggling to recruit and retain Latino participants in health research. The overall objective of this work is to generate a discussion about what we currently know about Latino response to the current US political culture, juxtaposed against well‐documented barriers to recruitment and retention and existing strategies for addressing these barriers. We propose that to overcome the lack of participation in health research by Latinos we must also be purposeful in how we address emerging concerns related to the current political incivility, or rather the current process of engaging in rude, insulting or threatening commentary or behaviors in political discussions or debates (Stryker, Conway, & Danielson, 2016). In this article, we submit that experiencing or witnessing political incivility, where one is a member of the group that is being targeted, is a form of discrimination and oppression. The purpose of this work is to illuminate how current political incivility towards Latinos deters their participation in research and uses a case study approach to illustrate how such comportment can have a negative impact on Latino‐focused research. The aims of the article are to: (a) describe how political incivility can serve as a proxy for discrimination; (b) discuss the impact of political incivility on health and health outcomes; (c) illustrate political incivility using a case example of how political incivility had an adverse impact on one research study on Latinos with diabetes and hypertension; and (d) present strategies for addressing challenges in Latino recruitment and retention. The topic is relevant and timely in light of the blatant political incivility in the pre‐ and postelection of Donald J. Trump, the current US president. Understanding the impact of the targeted incivility towards US Latinos is critical as several poor mental and physical health outcomes have been connected to experiences of discrimination (Abraído‐Lanza, Echeverría, & Flórez, 2016; Paradies, 2006; Williams, Neighbors, & Jackson, 2003). Furthermore, extant research suggests that experiencing discrimination may lead to limiting healthcare seeking behaviours or nonparticipation in activities that maintain health (Inzlicht, McKay, & Aronson, 2006; McSwan, 2000; Yoshikawa, Wilson, Chae, & Cheng, 2004).

## 2 POLITICAL INCIVILITY AND LATINO PERSPECTIVES ON CURRENT CONDITIONS

In a recent study on what constitutes political incivility, Stryker et al. (2016) found that over 82% of their respondents felt the use of slurs (racial, sexist, ethnic or religious slurs in a political discussion) as well as threatening or encouraging harm were highly politically uncivil. These types of statements and behaviours were overtly present in the political discourse of our then President‐elect Donald Trump. For example, Mr. Trump in a speech on 16 June 2015 stated of immigrants from Mexico, “They're bringing drugs. They're bringing crime. They're rapists. And some, I assume, are good people” (Lee, 2015). In addition to these stereotypes and generalizations, the Republican candidate made specific claims about specific immigration policies and enforcement that has been carried out to some extent under his subsequent victory. Mr. Trump campaigned heavily on promises to end illegal immigration through “building a wall,” strengthening border security and targeted deportation. President Trump has followed through on some of these promises, including signing an executive order to build (but not fund) a militarized wall and to add minor criminal infractions to deportable violations for undocumented immigrants (EO # 13767, White House Office of the Press Secretary, 2017). In some states and municipalities, local law enforcement has also agreed to collaborate with Immigration Control Enforcement (ICE), holding undocumented immigrants who interact with law enforcement until ICE can determine deportation status, expanding the reach of ICE efforts. Such action at the federal level has had an impacted on prior research efforts. For example, Vincent, McEwen, Hepworth, and Stump (2013) (encountered the impact of strict anti‐illegal immigration laws on recruitment in a study examining diabetes and Latinos. The passage of the law created a tense environment where potential and actual participants feared taking part in the study, thus having an impact on recruitment and retention.

## 3 CHALLENGES IN THE RECRUITMENT AND RETENTION OF LATINOS IN HEALTH RESEARCH

Better recruitment and retention of more representative participants in health research help ensure health care and treatment is appropriate and efficient across diverse groups. Even prior to an election where Latino immigrants were the target of political incivility, recruiting and retaining Latino people for research were difficult. Existing research on barriers to participation in health research points to several factors related to trust, risk and study design. As these are well established in the research, we will only visit these briefly and instead will focus most of our attention on traditional and innovative ways researchers are addressing these issues and how solutions may be created in light of the current uncivil political culture.

Research on recruitment and retention suggests that potential Latino participants express more concerns about researchers’ intentions, transparency and honesty about the true nature of the research project (García, Zuñiga, & Lagon, 2017; George, Duran, & Norris, 2013). Feelings of distrust not only keeps participant out of the research all together, but heightened suspicion may also increase hasty and premature exits even after recruitment has occurred. Current research also suggests that perceived risks for potential participants are pervasive and compelling. For instance, research participation may have an adverse impact on health insurance or legal status (George et al., 2013) and could potentially lead to deportation. Research has linked this fear to health indicators (Sheehan et al., 2016). Concerns about privacy (Sheehan et al., 2016) and protection of confidentiality (García et al., 2017) also loom for some potential and current participants. Others report fears about being further exposed to discrimination (García et al., 2017). In addition to typical study design issues that researchers face in working with marginalized population such as difficulty maintaining pathways for ongoing contact (García et al., 2017; Nguyen, Yan, Ell, Gonzalez, & Enguidanos, 2017), other challenges can emerge such as research staff not being properly trained or when the targeted recruitment of a particular minority group goes against their general feeling about fairness. In a recent study of principal investigators, research staff, referring clinicians and other health leaders Niranjan et al. (2017) found that the reluctance among some staff to recruit specifically on race was something that impeded their success in completing the study. Participants also noted a dearth in what they perceived as much needed cultural competency trainings in health research.

## 4 A CASE RESEARCH STUDY

A recent study (Benavides‐Vaello, 2017) serves as an example of how political incivility can have an impact on research and consequently have an impact on the overall health of Latinos in a state in the northwest. The intent of the exploratory descriptive pilot study was to identify the healthcare needs, in relation to diabetes and hypertension and associated risk factors, of Latinos in this state using a participatory action research approach that engaged religious institutions, safety net providers (Federally Qualified Health Centers [FQHCs]), members of the Hispanic community and other Hispanic service organizations. The knowledge gained from the pilot study was used to pursue an informed and focused translational clinical research grant to develop an intervention designed to address deficits identified in this Hispanic community. Qualitative and quantitative forms of inquiry were employed, and methods of data collection included individual interviews, focus groups as well as the administration of multiple surveys such as life quality, health literacy and discrimination. The pilot study involved the targeted recruitment of 50 Hispanic adult men and women residing in this northwestern state, with hypertension and/or diabetes.

The principal investigator (PI) was awarded funding (for 1 year) through the Mountain West Clinical and Translational Research Infrastructure Network (CTR‐IN) programme in July 2016. Funds were accessible by August 2016 and participant recruitment began in the same month. By this time period, candidates for the presidential election had been identified, with Hilary R. Clinton as the democratic candidate and Donald J. Trump the republican candidate. The presidential election was held on 8 November 2016. Between August 2016 and 5 November 2016, the PI was recruiting on average 10 participants per month (August, September and October). In early November, the PI was on course to meet the recruitment averages of prior months and anticipated completing recruitment, of 50 participants, by December 2016 or January 2017.

However, immediately after Donald J. Trump was elected into office, recruitment of participants for the pilot study dramatically dropped. Political incivility—particularly anti‐Latino and anti‐Muslim rhetoric—became commonplace and a more nationalistic sentiment grew. “Build the wall” became a widespread and openly shared mantra in demonstrations and political rallies that President‐elect Trump (and later confirmed as President Trump in January 2017) continued to hold. The PI reached out to community partners to inquire about support for recruitment, but health and social organizations working with Latinos in the state were also experiencing similar problems; that is, these individuals were in hiding and not maintaining appointments or taking part in outreach activities aimed at enrolling and reenrolling Latinos in health and social programmes. Religious institutions that were in the process of developing Latino outreach programmes, such as holding religious services in Spanish, also noted the drop‐in participation. Thus, there was an impact on another source of recruitment, as the PI had used Spanish religious services to make announcements about the study and had been successful in the past at identifying participants. Consequently, between 8 November 2016 and June 2017, only six additional participants were recruited (an average of one participant per month). Worth noting is that the majority of participants in the pilot study thus far were US citizens and/or legal residents. Thus, the impact of political incivility was not limited to undocumented Latinos in this state. What occurred in the study described here is aligned with other studies identified in the literature where national sentiment, in relation to anti‐immigration, had a negative impact on Latino participation in research (García et al., 2017; Martinez et al., 2015; Vincent et al., 2013). The PI has slowly overcome the impediments brought on by extant political incivility against US Latinos by returning to a word of mouth approach, maintaining values common to most Latino populations (discussed further in the following section) and sustaining consistent communication with Latino serving organizations to reaffirm the PIs presence in the community.

## 5 RECOMMENDATIONS AND STRATEGIES

As with barriers to recruitment and retention, researchers have also identified traditional and innovative ways to address these barriers in the health research field. In considering ways to build trust, reducing real and perceived risks and study design issues, we will also highlight how these may be adapted or strengthened with the current political incivility in mind.

### 5.1 Building trust and rapport

The most commonly identified way to reduce issues related to distrust was to find ways to incorporate personal contact and creating mutual trust through relationship‐building between the participants, research staff, family and peers (Ceballos et al., 2014; Díaz, Denner, & Ortiz, 2017; García et al., 2017; Sheehan et al., 2016) and through the use of brokers, community health workers or *promotores* (Díaz et al., 2017). We argue that this will continue to be a very important aspect of making health research accessible to Latino participants. Depending on the community‐level attitudes and behaviours towards Latino community members, the ability of outsiders to create rapport and trust may be extremely limited. If researchers are interested in doing health research in communities or states where police are collaborating with ICE in detaining suspected or confirmed undocumented immigrants, care must be taken to collaborate with trusted organizations such as faith communities and/or community‐based health centres. Researchers also need to conduct research that has cultural congruence and relatability (George et al., 2013) including what Díaz et al. (2017) call adhering to cultural scripts regarding *simpatia* (respectful interaction), *confianza* (support and trust) and *poderismo* (power of choice).

### 5.2 Reducing risks

Reducing real or perceived risks to important resources such as healthcare insurance or protected legal status as well as reducing the likelihood of further exposing participants to additional discrimination are of heightened prominence at this time in health research history. As researchers working with participants with increased vulnerabilities, it is particularly important to establish the limits of confidentiality at each institution and what legal steps law or immigration enforcement may take in pressuring principal investigators to release participant information. Once the realities of the legal limitations are established, these need to be communicated clearly for potential participants as part of informed consent. One way to reduce perceived risks and increase understanding is to use language that is most accessible for those being recruited. This is especially important in the initial contact, recruitment efforts and when explaining consent and confidentiality related to sensitive topics such as healthcare insurance or legal status. Research on language is mixed with some projects arguing that speaking Spanish increases participation and retention and others arguing language was not a particular barrier noted by their participants (Sheehan et al., 2016). From our assessment, determining the importance of language depends on the sought‐after population and their level of comfort with either dialect. Additionally, researchers must be flexible in their verbal and written language choices throughout the research process to adjust for unanticipated needs. For instance, Nguyen et al. (2017) had to make mid‐recruitment adjustments to their recruitment and consent processes after recognizing the need to have verbal materials in addition to written.

### 5.3 Study design, recruitment and implementation

In terms of research design, some research points to the value of community‐based participatory research and/or participatory action research (Choi, Heo, Song, & Han, 2016; Zhen‐Duan, Jacquez, & Vaughn, 2017) as a way to integrate the power of personal networks, develop in‐person trust and expectations about research staff cultural competencies. However, Choi and colleagues found this approach to be much more successful with their Korean participants when compared with those of Latino decent. Diaz, Denner, and Ortiz (2017) emphasize the need for a critical approach that acknowledges “context of current and past systems of opportunities and oppression” (p.155). In times where participants may feel particularly vulnerable to discrimination, researchers should be purposeful in their efforts to create space for open dialogue and questions (George et al., 2013; Sheehan et al., 2016) and in creating an environmental setting that is appropriate for the type of participation sought. For instance, Kanamori et al. (2016) were able to successfully create a space where Latina seasonal farmworkers could openly discuss sexuality, sexual activity and HIV prevention. Also related to study design and implementation, many recent scholars point to the importance of appropriate incentives (George et al., 2013; Rojas et al., 2016; Sheehan et al., 2016).

Study design and implementation should also take into consideration the contextual factors faced by potential participants. Latino participants tend to grapple with many competing demands on their time. Best practices suggest that participation in health research should be convenient and accessible (George et al., 2013). Researchers should consider issues such as location, timing, provision of appropriate child care and/or other services that will decrease the participant burden and increase the likelihood of inclusion. Another factor to consider during recruitment and in retention efforts is the living arrangements of the participant (Nguyen et al., 2017). These authors found that participants who were living in family households and stable living situations were more likely to enrol and stay in the study.

## 6 DISCUSSION

Increasing trust, developing a strong community presence, reducing risks (both real and perceived) and being thoughtful in research design and implementation, while considering critical contextual factors unique to being Latino in the USA are all important in improving recruitment and retention of this underrepresented group in health research. Nonetheless, these efforts are often more costly and require increased time and effort by the PI and/or research team. Consequently, added costs need to be incorporated into research budgets as does adequate time to develop those values important to Latino cultures (e.g., *simpatico*,* confianza*,* poderismo*) to produce high‐quality, inclusive research (Díaz et al., 2017).

## 7 CONCLUSIONS

Political incivility towards Latinos hinders their participation in research studies. The sequel of political incivility includes poorer mental and physical health outcomes for the targeted group, in this case Latinos. Additionally, political incivility further impairs the recruitment and participation of an already underrepresented group. Collectively, poorer health outcomes and decreased participation in research studies only contributes to the burgeoning burden of disease on low‐income Latinos. Diseases do not recognize citizenship status or political affiliations; thus, health disparities will worsen or remain a major health issue for one of our most vulnerable populations in this country. As researchers we must continue to use and find approaches that resonate with Latinos to increase study participation even during times of political incivility.

## CONFLICTS OF INTEREST

The authors declare no conflict of interest.
